# Feasibility and User Perception of a Fully Automated Push-Based Multiple-Session Alcohol Intervention for University Students: Randomized Controlled Trial

**DOI:** 10.2196/mhealth.3233

**Published:** 2014-06-23

**Authors:** Marcus Bendtsen, Preben Bendtsen

**Affiliations:** ^1^Technical FacultyDepartment of Computer and Information ScienceLinköping UniversityLinköpingSweden; ^2^Medical FacultyDepartment of Medical Specialist and Department of Medicine and HealthLinköping UniversityLinköpingSweden

**Keywords:** alcohol intervention, text messages, SMS, email, students, multiple-session intervention, push-based intervention

## Abstract

**Background:**

In recent years, many electronic health behavior interventions have been developed in order to reach individuals with unhealthy behaviors, such as risky drinking. This is especially relevant for university students, many of whom are risky drinkers.

**Objective:**

This study explored the acceptability and feasibility in a nontreatment-seeking group of university students (including both risk and nonrisk drinkers), of a fully automated, push-based, multiple-session, alcohol intervention, comparing two modes of delivery by randomizing participants to receive the intervention either by SMS text messaging (short message service, SMS) or by email.

**Methods:**

A total of 5499 students at Luleå University in northern Sweden were invited to participate in a single-session alcohol assessment and feedback intervention; 28.04% (1542/5499) students completed this part of the study. In total, 29.44% (454/1542) of those participating in the single-session intervention accepted to participate further in the extended multiple-session intervention lasting for 4 weeks. The students were randomized to receive the intervention messages via SMS or email. A follow-up questionnaire was sent immediately after the intervention and 52.9% (240/454) responded.

**Results:**

No difference was seen regarding satisfaction with the length and frequency of the intervention, regardless of the mode of delivery. Approximately 15% in both the SMS (19/136) and email groups (15/104) would have preferred the other mode of delivery. On the other hand, more students in the SMS group (46/229, 20.1%) stopped participating in the intervention during the 4-week period compared with the email group (10/193, 5.2%). Most students in both groups expressed satisfaction with the content of the messages and would recommend the intervention to a fellow student in need of reducing drinking. A striking difference was seen regarding when a message was read; 88.2% (120/136) of the SMS group read the messages within 1 hour in contrast to 45.2% (47/104) in the email group. In addition, 83.1% (113/136) in the SMS group stated that they read all or almost all the messages, compared with only 63.5% (66/104) in the email group.

**Conclusions:**

Based on the feedback from the students, an extended, multiple-session, push-based intervention seems to be a feasible option for students interested in additional support after a single-session alcohol intervention. SMS as a mode of delivery seems to have some advantages over email regarding when a message is read and the proportion of messages read. However, more students in the SMS group stopped the intervention than in the email group. Based on these promising findings, further studies comparing the effectiveness of single-session interventions with extended multiple-session interventions delivered separately or in combination are warranted.

## Introduction

Risky drinking among college and university students is a global problem that is a tremendous challenge to overcome by preventive measures [1]. In previous research, we highlighted the magnitude of risky drinking among Swedish students; we found repeatedly that at least 50% of students could be classified as risky drinkers [2,3]. Alcohol is known to be an important underlying factor for a substantial proportion of the global burden of disease [4,5].

Although brief face-to-face interventions delivered in various health care settings have been shown to be effective, implementation has been poor so far [6,7]. Because risky drinking is a major problem among students, there has been a call for more cost-effective interventions in order to reach large groups of students [8,9].

With the rapid development of the use of computers and the Internet, not least among students, a number of online alcohol interventions have been developed and evaluated in recent years. Systematic reviews provide some evidence of the effectiveness of online alcohol interventions targeted toward students [10-12]. However, there is great variety in the length and content of these online interventions from automated single sessions to online intervention provided at several time points during a semester [8,13-16].

Among the many challenges, online interventions that require participants to log on several times face a major challenge with compliance, for instance the participants do not use the intervention as intended. Thus, Web-based interventions where a person is guided to a Web page with reflective information, exercises, and home work to be done before logging in at a later stage have been shown to be difficult to implement, not at least in the area of alcohol interventions [17].

More recently, it has been suggested that various combinations of single-session interventions with email or SMS text messaging (short message service, SMS) might increase compliance [18-22]. This was also supported by a meta-analysis by Riper et al [9] who found that a significant difference was found between single-session, personalized, normative feedback and more extended Internet-based intervention.

Only a few studies so far have reported the use of emails as a part of an extended alcohol intervention. In a study by Moore et al [23] participants were interviewed for perceived barriers and acceptability of an extended alcohol intervention and it was found that SMS was preferred over email and Web-based methods. In other health behavior change areas, such as diet and physical exercise, the results so far appear promising for extended email interventions. Prompting by email to remind about self-monitoring of physical exercise was just as effective as prompting via email plus telephone [24]. Also, the Alive! email-based intervention for increasing physical activity and healthier diet was found to be effective compared with a wait-list control group. The intervention consisted of 25 personalized emails over a 3-month period [25].

In contrast to email-based extensions, previous research has, to a much larger extent, explored the use of SMS as part of an extended intervention, for example using SMS as reminders to log-on to a website or to perform self-monitoring of a health behavior [19-22]. In a review of 14 studies on behavior change interventions delivered by SMS messages, a positive outcome from the intervention could be measured in 13 studies [26]. However, participant retention ranged widely and in many studies at least 25% dropped out of the study. Also, the mode of initiating the intervention varied among studies and was found to be important for the effect of the intervention. Although the results were promising, a number of research issues were identified in order to learn how to optimize and enhance SMS message-based interventions [26].

An SMS-based alcohol intervention as a stand-alone intervention has so far only been tested in a few studies, however, promising results have been achieved in other areas such as tobacco use and weight loss [27,28]. One study using only SMS communication as an alcohol intervention showed promising results among young people discharged from an emergency department. Weekly assessment for 12 weeks followed by feedback were shown to increase reduction in alcohol consumption compared with a control group [29]. Another trial explored the perceived acceptability of adult trauma patients receiving SMS messaging as an aid to reduce harmful drinking behaviors. The patients recognized the potential benefit of such an intervention, although the study did not test whether a SMS-based intervention would be acceptable to the target group [30].

Two studies have explored the feasibility of collecting alcohol consumption data via SMS over a longer period of time, and compared the validity of the data with more traditional alcohol consumption questionnaires [23,31]. In both studies, SMS messages were found to be a valid source of data on consumption.

A few comparisons have been done between the use of SMS and email for an extended alcohol intervention. In a previous study, the Trial and Optimisation of Push-based High Alcohol Treatment 1 study (TOPHAT), we invited students participating in a single-session alcohol intervention to sign up for an extended push-based intervention over a number of weeks. The participants were given a choice of mode of delivery: SMS, email, or using an Android application and they could decide the length of the intervention (3-6 weeks) and the number of messages per week (3, 5, or 7 messages per week) [29]. Most students chose email as the mode of delivery; only 2.88% (33/1145) chose to download the Android application. In a follow-up immediately after the end of the intervention, no major difference was found concerning satisfaction with the mode of delivery, length of the intervention and frequency of messages. Overall, the participants (N=1138) who answered the follow-up questionnaire (response rate 82.68%, 941/1138) provided support for the feasibility and acceptability of a multiple push-based intervention delivered by SMS or email [32].

Since the effect size of single-session alcohol interventions is small, a large number of individuals who receive a brief Internet-based intervention continue to drink at levels that are considered risky [2,3,14,16]. Therefore, more developmental work and research is needed in order to optimize existing interventions or develop new means of communicating a health behavior change in order to accomplish an effect at the population level.

The present study, the TOPHAT-2 study, elucidates further on the feasibility of an extended push-based intervention by SMS or email to a nontreatment-seeking student population who have participated in a fully automated single-session alcohol intervention. Thus, the objective of the study was to compare the feasibility and user perception of an extended multiple-session intervention to students randomized to either an SMS or email push-based intervention.

## Methods

### Population and Recruitment

In mid-February 2013, all students on semesters 2, 4, 6, and 8 (a total of 5499 students) at the university in Luleå in northern Sweden were invited via their official university email address to complete a fully automated single-session online alcohol intervention by clicking on an embedded link in the email. The content of the invitational email and the single-session intervention was similar to that used in routine practice at universities throughout Sweden, as reported previously [2,3,16]. The only additional information was an offer to join a research project after having received the usual three pages of feedback from the single-session intervention. The invitation was signed both by the director of the local student health care center and the research leader (PB). After 1 and 2 weeks, a reminder was sent to those who had not completed the single-session intervention, and after 3 weeks the questionnaire was closed and no more responses were possible. The students completed the single session on a computer, smart phone, or tablet at their own convenience. After having completing the single session screening, all participants received personalized feedback direct on their computer and was also mailed a copy of the feedback.

**Figure 1:**
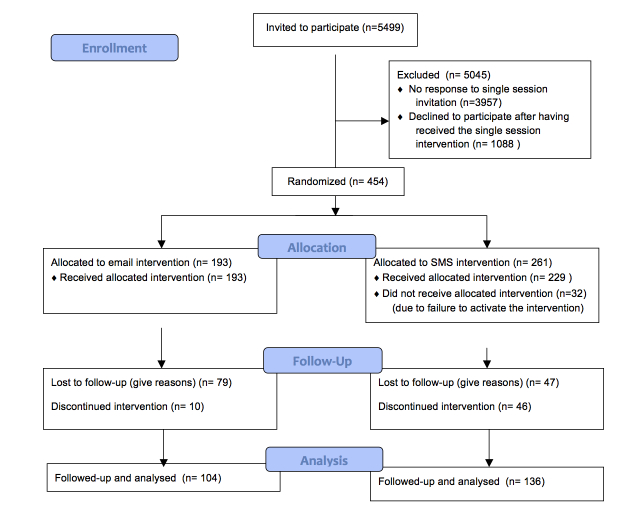
Consort flow chart.

After completing the single-session intervention (screening and personalized feedback), the students were offered participation in a draw for an iPad if they were willing to take part in an additional extended intervention after the single session, as part of a research project. Only participants who answered the follow-up questionnaire were included in the draw. All students, regardless of alcohol consumption, were invited to join the research project and participate in the extended intervention. The students were told that they would be randomized to either an SMS or email intervention. No other means of registering for the extended intervention was made available. An overview of the recruitment process and study design is shown in Figure 1.

### Signing Up and Completion of the Extended Intervention

All participants interested in joining the extended intervention were asked to submit their telephone number. They were then randomly assigned to receive the extended intervention via email or SMS. Randomization was done using Java’s built in random number generator (java.util.Random). Randomization was thus fully computerized, did not use any strata or blocks, and could not be subverted because this and all subsequent study processes were fully automated (programmed by MB).

Initially, the participants were invited via email to complete the single-session intervention, therefore no further steps were necessary for those randomized to email because we already had their email address. However, all participants who were assigned to SMS were asked to confirm their participation by responding to an initial SMS in order to ensure that the telephone number was correct.

Thirty-two of the students who were randomized to the SMS intervention (n=261) did not manage to activate the intervention (ie, gave an incorrect telephone number, did not respond to the initial SMS or typed the wrong confirmation word) and therefore never received the intervention. This lead to a study group of 422 participants, with 229 participants assigned to the SMS group, and 193 to the email group (Figure 1).

Once a participant was randomized (and for the SMS group having confirmed the telephone number) the intervention ran for 4 weeks. All participants received the same intervention content. The participants could terminate the intervention at any time by sending an SMS with the text “Stop” or answering an email with the word “Stop.” After the participant received the last message, an email was sent containing a link to the follow-up questionnaire. The follow-up questionnaire was only sent to those completing the intervention and therefore those who actively terminated the intervention (n=56) did not receive the follow-up questionnaire.

### Content of the Extended Intervention

The new extended intervention consisted of messages delivered to participants at a specific time during the week. There were four messages per week, for a total of four weeks.

The textual content of the messages was created based on prevailing theories within the field of behavior change, including Self-Determination Theory, Social Cognition Models, Social Cognitive Theory, Theory of Planned Behavior, and Model of Action Phases. The messages were also inspired by some of the assessment questions commonly used in alcohol research. The content of the messages was labeled in order to keep track of when a particular type of message was sent. The labels used for the construction of the messages schedule were “food for thought,” “task,” “challenges,” and “reflective.” Examples of these messages are outlined in Textbox 1.

To allow participants to get a current assessment of their alcohol consumption, a direct link was enclosed to the study’s home page after each message, where more information on safe drinking limits was available. In addition, a direct link was provided to the same Web-based single-session alcohol assessment as used in the baseline assessment.

**Examples of messages sent to motivate less drinking.**

Examples of “food for thought” messages:

What are the most important things in your life? How does drinking affect them?

How convinced are you that you are OK with your alcohol habits?

Examples of “task” messages:

List three good things and three not so good things about your drinking.

Think about a recent situation where you drank more alcohol than you indented. Formulate a sentence that starts with “what if… then I would not have been in that situation. Ask yourself how you could avoid similar situations in the future.

Examples of “challenge” messages:

Tonight or next time you are going out for a drink – decide to take a glass a water between every drink. This will make you feel better the next day – and you will probably save some money.

Tonight or next time you are going out for a drink – decide before you start drinking how much you are going to drink – and try to keep track and stick to this goal during the evening. If you fail to stick to your own goal – think about what went wrong?

Examples of “reflective” messages:

Is the way that you drink fully in accordance with your own values?

When you get time, now or perhaps later, consider how you normally feel the day after you have been drinking. Is there anything that you are dissatisfied with?

### Extended Intervention Message Schedule

The messages were sent out on Wednesdays, Fridays, Saturdays, and Sundays. “food for thought” messages were sent on Wednesdays, “tasks” were sent on Fridays, “challenges” on Saturdays and “reflective” messages on Sundays.

### Measurements

#### Risky Drinking at Baseline

Risky drinking was defined according to the official definition used in Sweden, which includes two criteria: total weekly consumption, and frequency of heavy episodic drinking. Risky total weekly consumption of alcohol was defined as drinking more than 9 (females) or 14 (males) standard drinks/units per week (1 standard unit = 12 g of alcohol; eg, a small glass of wine). Heavy episodic drinking was defined as drinking more than 4 (females) or 5 (males) standard units on a single occasion (eg, during an evening). Having one or more episodes of heavy drinking per month was considered risky drinking. Participants were considered risky drinkers if they fulfilled either or both of the definitions described above. These drinking limits for safe drinking are the official limits used in Sweden [33].

Weekly alcohol consumption was calculated by adding the number of standard drinks consumed during the last 7 days. Heavy episodic drinking was assessed by responding to how often the participants drank 5 (men)/4 (women) or more standard drinks on a single occasion with the following response options: less than once a month, once a month, 2 to 3 times a month, once a week, or 2 or more times a week.

Motivation to change was assessed with the following response options: “I have not thought about decreasing my consumptions,” “I have thought about decreasing my consumption, but I am not thinking about it right now,” “I am thinking about decreasing my consumption,” “I have started to decrease my consumption,” “ I have tried to decrease my consumption, but failed.”

#### Perceived Drinking Compared With Peers at Baseline

Students were asked if they thought they drank “more,” “less,” or “the same” as their peers as part of the assessment in the single-session intervention. This was used in the analysis of the feasibility evaluation for the extended intervention. In the single-session feedback, the students were shown a graphic comparison between their actual consumption compared with peers of the same age group and sex. The comparison was based on a reference database held by the authors from the previous 5 years of surveys completed throughout Sweden, consisting of more than 200,000 measurements on students.

### Follow-Up Questionnaire

The follow-up questionnaire comprised 12 questions exploring the feasibility and usefulness of the extended intervention as perceived by the students. Two questions explored whether the student had changed their alcohol consumption and the reasons for a reduction in consumption: satisfaction with the length of the intervention period (too long, just right, too short, don’t know), and the number of messages (too many, just right, too few, don’t know).

One question explored satisfaction with the delivery method.The participants were told that the messages could be received via SMS or email. The response options were “Yes I was satisfied with the delivery method,” or “I would have preferred the other delivery method.” One question explored the estimated proportion of messages read (all, nearly all, most of the messages, about half, a few, very few, none). Another question asked the participants to estimate the average time it took before a message was read (immediately, within 1 hour, within 3 hours, the same day, next day, several days later, read almost none of the messages).

One question explored the students’ overall perception of the content of the messages (very good, good, poor, very poor). Two questions explored the proportion of messages that the participants considered to be good, useful or bad, not useful (all, nearly all, most of the messages, about half, a few, very few, none).

The last questions asked whether the student would recommend the intervention to a friend who drinks too much (yes definitely, possibly, doubtful, don’t know). The participants could also comment on their responses to each question and give an overall comment on the intervention. The study was approved by the regional ethical committee in Linköping, Sweden (no. 2013/94-31).

### Statistical Analysis

All data from the single-session assessment were used to characterize the students using the following variables: age, sex, social status, semester, perceived drinking compared with peers, motivation to change, and risky drinking status. Differences between students within different response options to the questions in the follow-up questionnaire were examined using chi-square tests without any adjustment for baseline characteristics. In cases where cell values were too small for reliable chi-square output, the Fisher exact test was used. If cell values were too small for both the chi-squared and Fisher tests, an attempt was made to pool the variables. Only tests where *P*<.05 were considered. All statistical analyses were performed using R version 2.15.1.

## Results

### Response Rate and Characteristics of the Participants

Among the 5499 students who were invited to participate in the first stage of the study, 1542/5499 (28.04%) completed the single-session intervention and received feedback. The initial single-session intervention was sent to all students on semesters 2, 4, 6, and 8 at Luleå University using the official university mailing list and therefore we did not know the age, sex, and social status of the total population of the students invited to participate. However, the baseline characteristic of the students who agreed to participate in the extended session intervention (n=454) compared with those declining participation (n=1088) did not differ significantly, except for motivation to change; more participants than nonparticipants had started to decrease their consumption (100/454, 22.0% vs 167/1088, 15.35%) (Table 1).

The characteristics of the participants are presented in Table 2. No significant differences between the groups were seen. Approximately one-half of the participants in both groups were risky drinkers.

**Table 1:** Baseline characteristics of the participants who agreed to participate or declined participation in the extended intervention (*N*=1542).

		Participated n=454 (%)	Did not participate n=1088 (%)	χ^2^(df)	*P* value
**University semester**				3.63 (3)	.588
	2	186 (40.97)	469 (43.11)		
	4	109 (24.01)	276 (25.37)		
	6	112 (24.67)	236 (21.69)		
	8	47 (10.35)	107 (9.83)		
**Sex**				0.9 (1)	.342
	Female	218 (48.02)	553 (50.83)		
	Male	236 (51.98)	535 (49.17)		
**Age**				2.48 (3)	.289
	18–20 years	81 (17.84)	198 (18.20)		
	21–25 years	265 (58.37)	670 (61.58)		
	26–30 years	69 (15.20)	125 (11.49)		
	31+ years	39 (8.59)	95 (8.73)		
**Social status**				0.37 (1)	.542
	No partner	234 (51.54)	581 (53.40)		
	Have a partner	220 (48.46)	507 (46.60)		
**Motivation to change** ^a^					.019^b^
	I have tried to decrease my consumption, but failed	1 (0.26)	6 (0.65)		
	I am thinking about how to change my habits	9 (2.34)	30 (3.23)		
	I have thought about changing, but I`m not thinking about it right now	37 (9.64)	107 (11.52)		
	I have started decreasing my consumption	100 (26.04)	167 (17.97)		
	I have not had any thoughts regarding change	237 (69.53)	619 (66.63)		
**Risky drinking**				0.32 (1)	.570
	No	232 (51.10)	537 (49.36)		
	Yes	222 (48.90)	551 (50.64)		

^a^Only includes students who had been drinking in the previous 3 months.

^b^Fisher exact test.

**Table 2:** Characteristics of the students in the SMS (N=229) and email groups (N=193).

			Email group (N=193)	SMS group (N=229)	χ^2^(df)	*P* value
			n (%)	n (%)		
**Sex**					0.41 (1)	.524
	Female		90 (46.6)	115 (50.2)		
	Male		103 (53.4)	114 (49.8)		
**Age**					0.57 (3)	.904
		18-20 years	35 (18.1)	38 (16.6)		
		21-25 years	112 (58.1)	134 (58.5)		
		26-30 years	28 (14.5)	38 (16.6)		
		31+ years	18 (9.3)	19 (8.3)		
**Social status (pooled data)**					0 (1)	.960
		No partner	100 (51.8)	117 (51.1)		
		Have a partner	93 (48.2)	112 (48.9)		
**Perceived drinking compared with peers (pooled data)**					2.32 (2)	.313
		More	25 (12.9)	22 (9.6)		
		Same	49 (25.4)	52 (22.7)		
		Less	119 (61.7)	155 (67.7)		
**Motivating to change (pooled data)** ^a^					2.3 (2)	.317
		No thoughts of change	95 (57.9)	124 (64.6)		
		Thought of change	19 (11.6)	23 (12.0)		
		Taken action	50 (30.5)	45 (23.4)		
**Risky drinking**					2.03 (1)	.154
		No	91 (47.2)	125 (54.6)		
		Yes	102 (52.8)	104 (45.4)		

^a^Only includes students who had been drinking in the previous 3 months.

### Drop-Out During the Extended Intervention and Response Rate to the Follow-Up

During the extended intervention, 10/193 participants (5.2%) in the email group asked to stop the intervention and 46/229 participants (20.1%) in the SMS group asked to stop the intervention (χ^2^_1_=20.22, *P*<.001). There were no significant differences between those who asked to stop the intervention for any of the baseline characteristics such as semester, sex, age, social status, drinking compared with peers, motivation to change or risk drinking status.

A total of 240/454 follow-up questionnaires were returned giving a response rate of 52.9%. There were no significant difference in the response rate between the two groups; 53.9% (104/193) responded in the email group and 52.1% (136/261) in the SMS group (χ^2^_1_=0.14, *P*=.700).

All questions had to be completed. Therefore, no internal data were missing for any of the questions (Figure 1).

### Main Findings

Satisfaction with the length of the intervention and number of messages per week did not differ between the SMS and email groups (Table 3). Only 4.2% (10/240) thought that the intervention was too long but around 72.9% (175/240) found the length just right. Of the participants, 57.9% (139/240) were satisfied with the number of messages per week, but 42.1% (101/240) thought that there were too many messages. Only 4 participants thought that there were too few messages (Table 4). There were no differences in satisfaction with the frequency of messages and the number of messages per week with regards to baseline characteristics, such as sex, age, risk drinking status, perceived drinking compared with peers, or motivation to change.

There were 84.6% (203/240) of the participants who found the overall content of the intervention to be good or very good, and 48.8% (117/240) perceived that all or almost all of the specific messages were good. No differences were seen with regard to mode of delivery or any of the baseline characteristics except that participants with risky drinking at baseline were less satisfied with the content than nonrisky drinkers (74.0%, 54/73 vs 89.2%, 149/167; *P*=.015, Fisher exact test).

We asked the participants whether they would recommend the intervention to a friend who needed to cut back on their alcohol consumption. The intervention would definitely be recommended by 30.4% (73/240), 34.6% (83/240) would possibly recommend it, 24.2% (58/240) were doubtful, and 2.5% (6/240) did not know. No difference was seen between risky and nonrisky drinkers or by mode of delivery. Of the participants, 80% (192/240) were satisfied with the mode of delivery offered to them, but 14.0% in the SMS group (19/136) and 14.4% (15/104) in the email group would have preferred the other delivery method and 5.8% (14/240) did not know which method they preferred.

**Table 3:** Satisfaction with the length of the intervention in relation to background characteristics (n=240).

		Satisfaction with the length of the intervention				χ^2^(df)	*P* value
		Too long, n (%)	Just right, n (%)	Too short, n (%)	Don’t know, n (%)		
**University semester**							
	2	3 (2.7)	85 (76.6)	16 (14.4)	7 (6.3)	6.82 (9)	.656
	4	1 (2.0)	38 (76.0)	6 (12.0)	5 (10.0)		
	6	5 (8.5)	39 (66.1)	8 (13.6)	7 (11.9)		
	8	1 (5.0)	13 (65.0)	4 (20.0)	2 (10.0)		
**Sex**							.259^b^
	Female	2 (1.8)	85 (78.0)	13 (11.9)	9 (8.3)		
	Male	8 (6.1)	90 (68.7)	21 (16.0)	12 (9.2)		
**Age**							.877^b^
	18-20 years	2 (4.7)	31 (72.1)	7 (16.3)	3 (6.9)		
	21-25 years	5 (3.5)	103 (72.5)	21 (14.8)	13 (9.2)		
	26-30 years	2 (5.6)	25 (69.4)	4 (11.1)	5 (13.9)		
	31+ years	1 (5.3)	16 (84.2)	2 (10.5)	0 (0.0)		
**Perceived drinking compared with peers (pooled data)**							.282^b^
	More	1 (3.2)	20 (64.5)	4 (12.9)	6 (19.4)		
	Same	4 (7.7)	37 (71.2)	6 (11.5)	5 (9.6)		
	Less	5 (3.3)	114 (74.5)	24 (15.7)	10 (6.5)		
**Motivation to change (pooled data)** ^a^							.146^b^
	No thoughts of change	5 (4.1)	88 (72.1)	21 (17.2)	8 (6.6)		
	Thoughts of change	0 (0.0)	23 (88.5)	2 (7.7)	1 (3.8)		
	Taken action	4 (7.4)	35 (64.8)	6 (11.1)	9 (16.6)		
**Risky drinking**							.509^b^
	No risk	3 (2.6)	85 (73.3)	19 (16.4)	9 (7.7)		
	Yes risk	7 (5.6)	90 (72.6)	15 (12.1)	12 (9.7)		
**Mode of delivery**							.329^b^
	Email	4 (3.9)	74 (71.2)	13 (12.5)	13 (12.5)		
	SMS	6 (4.4)	101 (74.3)	21 (15.4)	8 (5.9)		

^a^Only included students who had been drinking in the previous 3 months.

^b^Fisher exact test.

**Table 4:** Satisfaction with the number of messages per week in relation to background characteristics.

		Satisfaction with number of messages per week				*P* value^b^
		Too many, n (%)	Just right, n (%)	Too few, n (%)	Don’t know, n (%)	
**University semester**						
	2	40 (36.0)	67 (60.4)	1 (0.9)	3 (2.7)	.362
	4	17 (34.0)	30 (60.0)	3 (6.0)	0 (0.0)	
	6	25 (42.4)	33 (55.9)	0 (0.0)	1 (1.7)	
	8	11 (55.0)	9 (45.0)	0 (0.0)	0 (0.0)	
**Sex**						.537
	Female	38 (34.9)	68 (62.4)	1 (0.9)	2 (1.8)	
	Male	55 (41.9)	71 (54.2)	3 (2.3)	2 (1.5)	
**Age**						.400
	18-20 years	14 (32.6)	25 (58.1)	2 (4.6)	2 (4.6)	
	21-25 years	56 (39.4)	83 (58.5)	1 (0.7)	2 (1.4)	
	26-30 years	17 (47.2)	18 (50.0)	1 (2.8)	0 (0.0)	
	31+ years	6 (31.6)	13 (68.4)	0 (0.0)	0 (0.0)	
**Perceived drinking compared with peers (pooled data)**						.814
	More	13 (41.9)	17 (54.8)	1 (3.2)	0 (0.0)	
	Same	22 (42.3)	30 (57.7)	0 (0.0)	0 (0.0)	
	Less	57 (37.3)	89 (58.1)	3 (1.9)	4 (2.6)	
**Motivation to change (pooled data)** ^a^						.108
	No thoughts of change	52 (42.6)	67 (54.9)	1 (0.8)	2 (1.6)	
	Thoughts of change	5 (19.2)	19 (73.0)	1 (3.9)	1 (3.9)	
	Taken action	21 (38.9)	31 (57.4)	2 (3.7)	0 (0.0)	
**Risky drinking**						.417
	No risk	67 (40.1)	94 (56.3)	2 (1.2)	4 (2.4)	
	Yes risk	26 (35.6)	45 (61.6)	2 (2.7)	0 (0.0)	
**Mode of delivery**						.216
	Email	41 (39.4)	60 (57.7)	0 (0.0)	3 (2.9)	
	SMS	52 (38.2)	79 (58.0)	4 (2.9)	1 (0.7)	

^a^Only included students who had been drinking in the previous 3 months.

^b^Fisher exact test.

### Number of Messages Read and the Timing

The participants in the SMS group reported reading more of the messages than the participants in the email group. Thus, 55.9% in the SMS group (76/136) stated they had read all messages versus 42.3% (44/104) in the email group and 27.2% (37/136) versus 21.2% (22/104) stated that they had read almost all (*P*=.030, Fischer exact test).

When the messages were read also differed between the SMS group and the email group. In the SMS group, 51.5% (70/136) read most of the messages immediately on receipt; in contrast, only 22.1% (23/104) in the email group read the messages at once. In the SMS group, 88.2% (120/136) of the participants read the messages within 1 hour in contrast to 45.2% (47/104) in the email group. In the email group, 19.3% (20/104) read the messages the next day or later, whereas none did so in the SMS group.

### Change in Alcohol Consumption

Although the study did not aim to evaluate the actual effect of the messages on alcohol consumption, we did ask the participants to estimate any potential change in their alcohol consumption during the last 2 months. Twenty-three percent (55/240) stated that they had decreased their consumption (21.1%, 23/109 among females and 30.5%, 40/131 among males). In the youngest age group (18-20 years), a larger proportion (37.2%, 16/43) reported a decrease in consumption compared with 25.4% (36/142) among those aged 21 to 25 years and 20% (11/55) in those aged 26+ years (χ^2^_8_=16.03, *P*=.042). However, no significant difference in reduction of alcohol consumption was seen between the two modes of delivery.

Those stating that they had decreased their consumption (n=63) were asked to evaluate with a yes/no response if the messages contributed to the reduction in alcohol consumption. Thirty-seven stated that the messages did not contribute to the reduction, whereas 41% (26/63) agreed that they did. Among those who received the messages via email, 20.8% (5/24) stated that the messages contributed to a reduction in contrast to 53.8% (21/39) in the SMS group (χ^2^_1_=5.39, *P*=.020).

## Discussion

### Principal Findings

This study explored the feasibility and user perceptions of an extended alcohol intervention to students who had participated in a fully automated online single-session alcohol intervention. The students were randomized to receiving the intervention either by SMS or email. To our knowledge, this is the first study to compare two modes of delivery of an extended intervention using the same messages.

No differences between the two modes of delivery were seen with regard to satisfaction with the length of the intervention and the number of messages per week. In this study, the length of the intervention was 4 weeks for all participants and only 4.2% (10/240) of the participants thought that this was too long. On the other hand 42.1% (101/240) of the participants thought that the number of messages per week was too many (four messages per week).

In a previous study, we gave the participants a choice on the length of the intervention and the number of messages per week and we found that most participants chose the shortest length (3 weeks) and the lowest number of messages per week (3 per week) [32]. Satisfaction with the length of the intervention in the present study was higher than in the previous study indicating that the participants are somewhat hesitant to sign up for a longer intervention. No difference in satisfaction was seen with regard to the length of the intervention and the number of SMS messages among risky and nonrisky drinkers. Risky drinkers would likely have to receive a longer intervention. In a recent study on perceived acceptability of an SMS intervention, concern was raised about receiving messages to often without specifying the frequency [30]. In another study in university students of SMS messages as surveillance of students, an SMS per day was acceptable for most participants, and the participants perceived SMS messages as a more positive means of delivery, compared with telephone calls or email [23]. Further studies needs to explore the optimal balance between what is needed in order to support a behavior change and what is acceptable for the target population.

We found no difference between the SMS and email groups with regard to perceived satisfaction with the content of the intervention, which in general was perceived as good or very good. This was similar to our previous study where the participants could choose the mode of delivery [32].

Importantly, most of the participants were satisfied with the mode of delivery given to them and around 14.2% (34/240) in both groups would have preferred the other mode of delivery. This should be seen in the light of our previous study; the students were given a free choice between email, SMS, and an Android application and 83% (952/1145) chose email [32]. In the present study, we noticed a higher dropout rate in the SMS group (20.1%, 46/229) compared with the email group (5.2%, 10/193), which is difficult to explain because we did not see any difference in the baseline characteristics between the dropouts and those who remained in either of the groups. However, this dropout rate is in parity with a number of previous studies [26]. One reason for the higher dropout rate in the SMS group could be that SMS messages were perceived as more intrusive; the mobile phone likely gave an incoming SMS alert each time a new message was received and this may have led to some inconvenience for the students. The need for careful timing of the messages was also emphasized in a study on university students who did not want a message too early in the morning [23] and in another study, messages during dinnertime were perceived as potential annoying [30]. On the other hand, students receiving emails could also have perceived the messages as intrusive because an incoming email might also give rise to an alert. Future studies needs to take this into account.

In the present study, we added some additional explorative follow-up questions such as the self-reported number of messages read and the timing of reading the messages. We noted that students receiving the messages via SMS read more of the messages compared with the email group. The timing for receiving challenging information should be right (ie, just before going out for a drink on a Friday evening). Thus, 88.2% (120/136) of the participants in the SMS group read the messages within 1 hour, but only 45.2% (47/104) of the participants in the email group. In a review on SMS messages for behavioral change, it was highlighted that there is a lack of process studies exploring how messages are treated and stored by the user [26]. Our results contribute somewhat to our understanding that SMS messages are more likely to be read relatively soon after receipt, in contrast to email. This study consider calculating the cost of sending mail to a group of students compared with the cost of sending SMS. Pending on local technical solutions the cost may be comparable, which was the case in the present study since we were able to set up a technical solution with minimal cost. However, if an SMS intervention has to be administrated by an external commercial company the cost for SMS may be considerably more than for an email intervention.

On a more subjective note, 54% (21/39) of those in the SMS group that had decreased their alcohol consumption stated that the messages had helped them in their effort, in contrast to only 21% (5/24) in the email group. Although encouraging, this finding needs to be explored further in a forthcoming randomized controlled trial.

### Limitations

The study was performed in an unselected group of students primarily not seeking help for their alcohol consumption and including both risky and nonrisky drinkers. We also introduced a bias when offering the participants entry to a draw for an iPad. However, from previous studies, we know that this helps in getting a sufficient number of participants, which we decided would be acceptable in this explorative study. This means that the results should be taken with some reservations. Still, the purpose of this explorative study was to get an idea of what is feasible among students in order to get good compliance with the intervention. Single-session and extended interventions will always include individuals with a strong motivation as well as less strong motivation and preferably should satisfy both groups.

Due to the larger drop-out rate in the SMS group than in the email group 20.1%, (46/229) versus 5.2% (10/193) we cannot be sure that the apparently equal satisfaction with the intervention in both groups reflects the opinion of all who signed up for the intervention. There might be a greater dissatisfaction with the intervention when considering all who signed up for the SMS intervention. However, we do not have data about satisfaction on those who dropped out.

The response rate to the initial single-session intervention was 28.04% (1542/5499), which is somewhat lower than in our previous studies [2,3,16]. Also, despite offering the participants to participate in a draw of an iPad if they completed the follow-up questionnaire, the follow-up rate in the present study was only 52.9% (240/454), which was lower than in the previous study (82.7%) [32]. The two studies were performed at two different universities in Sweden, which could partly explain the difference in the response rate.

Participation was offered to both risky and nonrisky drinkers since we wanted as many views as possible on the structure and content of the extended intervention. The proportion of participants signing up for the extended intervention was equally distributed with regard to all baseline characteristics (Table 1) and between the participants randomized to the SMS and email groups (Table 2).

### Conclusions

Based on feedback from the students, an extended push-based intervention delivered via SMS or email seems to be feasible to offer those interested in additional help after a single-session intervention This is further emphasized by the large proportion of students who would recommend the intervention to a friend needing to cut back on their drinking. The perception of the intervention did not differ between mode of delivery with regards to the length of the intervention, the number of messages per week and overall satisfaction with the given mode of delivery.

However, the number of messages read and the timing of reading them differ between the SMS and email groups. This may indicate that SMS is more effective in delivering messages as intended (eg, when sending challenges that should be read in real time) say on a Friday night before starting drinking. On the other hand, more students dropped out in the SMS arm. How best to get participants to stay with the intervention as well as ensuring that the messages are read at the intended time is something that needs to be explored in future studies.

In a forthcoming randomized controlled trial, based on the findings in the present study, we are now confident that a realistic comparison will be to study the effectiveness of a single-session intervention with an extended SMS or email intervention, comparing these in separate arms and in a combined arm.

## Abbreviations

SMS: short message service
TOPHAT: Trial and Optimisation of Push-based High Alcohol Treatment
